# Phytochemical characterization and bioactivity evaluation of *Artemisia abyssinica* methanolic extract as a baseline for bioresource exploration

**DOI:** 10.1186/s40643-026-01103-7

**Published:** 2026-07-20

**Authors:** Abdu Aldarhami, Suliman A. Alderhami, Hassen Harzali, Abdulrahman S. Bazaid, Zarah I. Alzahrani, Hassan A. El-Adawy, Nashwa H. Abdullah, Nizar H. Saeedi, Khaled A. Abdelshafeek, Ahmed A. Elhenawy

**Affiliations:** 1 Department of Microbiology and Parasitology, Faculty of Medicine, Umm Al-Qura University, Al-Qunfudhah, Saudi Arabia; 2 Department of Chemistry, Faculty of Science, Al-Baha University, Al-Baha, Saudi Arabia; 3 Applied Mineral Chemistry Laboratory (LR19ES02), Department of Chemistry, Faculty of Sciences of Tunis, University of Tunis El Manar, Tunis, Tunisia; 4 Department of Medical Laboratory Science, College of Applied Medical Sciences, University of Ha’il, Hail, 55476 Saudi Arabia; 5 Department of Chemistry, Faculty of Science, Al- Azhar University, Nasr City, Cairo 11884 Egypt; 6 Botany and Microbiology Department, Faculty of Science, Helwan University, Cairo, Egypt; 7 Department of Medical Laboratory Technology, Faculty of Applied Medical Sciences, University of Tabuk, Tabuk, Saudi Arabia; 8 Pharmaceutical Industries and Drugs Institute, Chemistry of Medicinal Plants Department, National Research Centre, El Bohouth St, Dokki, Giza 12622 Egypt

**Keywords:** *Artemisia abyssinica*, Phytochemical profiling, Phenolic compounds, Flavonoids, LC–MS/MS, GC–MS, DPPH assay, Antibacterial activity, Antioxidant activity, Molecular docking

## Abstract

The growing prevalence of antimicrobial resistance and cancer has intensified interest in natural products as sources of biologically active compounds with potential pharmaceutical relevance. In this study, the phytochemical composition of the methanolic extract of *Artemisia abyssinica* aerial parts (MEAA) was comprehensively characterized using liquid chromatography–tandem mass spectrometry (LC–MS/MS) and gas chromatography–mass spectrometry (GC–MS), and its in vitro antimicrobial, antioxidant, cytotoxic, and molecular docking properties were evaluated. LC–MS/MS analysis revealed a diverse profile dominated by flavonoids and phenolic compounds, including myricetin, quercetin, apigenin derivatives, hispidulin, and taxifolin, while GC–MS analysis tentatively identified several non-polar constituents, with gitoxigenin as a putative major component. MEAA exhibited moderate antibacterial activity against *Micrococcus luteus* (inhibition zone 15 mm; MIC = 2 mg/mL) and moderate activity against *Streptococcus pneumoniae* and *Escherichia coli*. The extract demonstrated weak antioxidant activity in the DPPH assay (IC_50_ = 268.15 µg/mL) and moderate cytotoxicity against HepG-2 hepatocellular carcinoma cells (IC_50_ = 59.2 µg/mL) compared with Adriamycin (IC_50_ = 6.9 µg/mL). Molecular docking was employed to provide supportive structural insights, suggesting that selected flavonoids, particularly myricetin, apigenin, and hispidulin, may interact favorably with DNA gyrase, epidermal growth factor receptor, and urease. Overall, this study provides an analytical and biological profile of MEAA and highlights its phytochemical richness and early-stage bioactivity, supporting its relevance for further pharmaceutical investigation as well as its potential as a natural bioresource for future applications.

**Graphical abstract:**

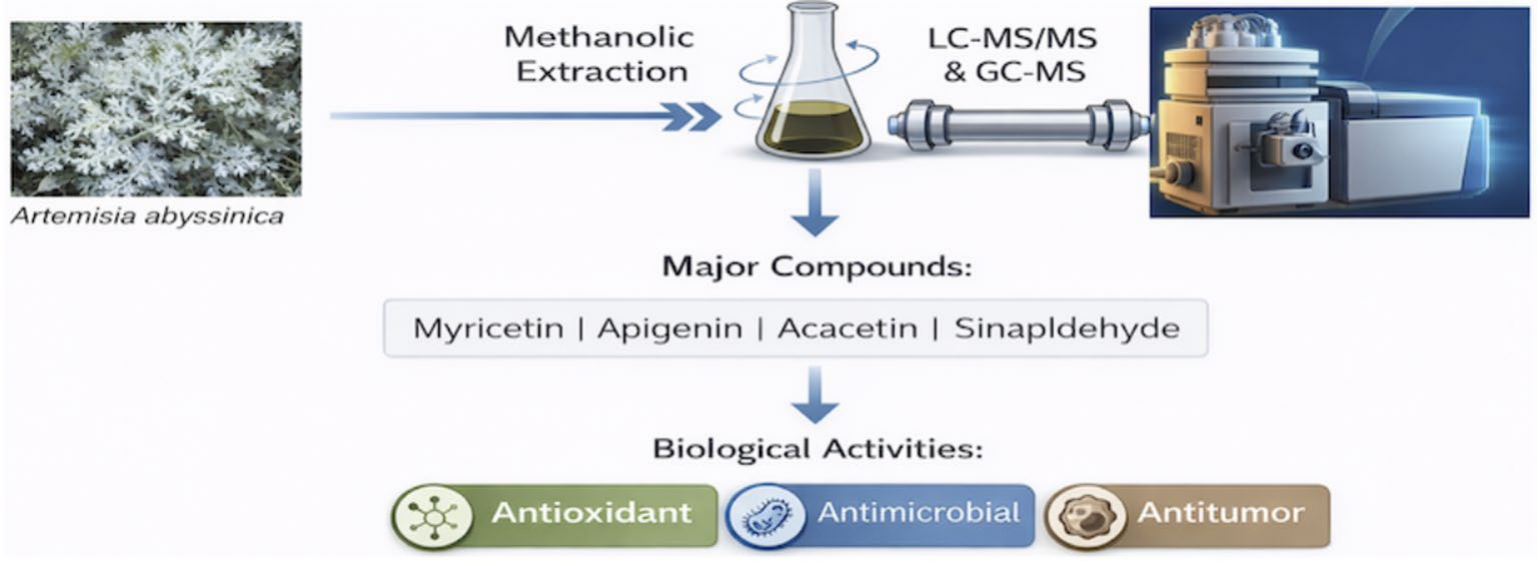

## Introduction

Infectious diseases remain a major cause of global morbidity and mortality (Singh et al. 2023). With the increase in drug-resistant pathogens, there is growing interest to develop new methods for the prevention and treatment of infections using phytochemical compounds derived from medicinal plants. Such natural products have inspired many therapeutically effective drugs; however, only a limited number of plant extracts have undergone rigorous safety and efficacy evaluations in humans or animals (Singh et al. 2023). In this context, medicinal plants continue to attract clinical interest as potential sources of bioactive compounds that may contribute to early-stage antimicrobial and anticancer drug discovery (Singh et al. 2023). In addition to their therapeutic relevance, medicinal plants represent important renewable bioresources that can be explored for the development of value-added bioactive compounds.

Cancer is a multifactorial disease involving genetic and epigenetic alterations that drive uncontrolled cell proliferation and invasion. Oxidative stress and redox dysregulation are implicated in tumor initiation and progression, and the chemical diversity of medicinal herbs continues to provide candidates for anticancer investigation (Kooti et al 2017, Atanasov et al 2015).

The family Asteraceae, comprising approximately 1540 genera and 23,000 species, is among the largest dicot flowering plant families. Members are distributed across diverse ecosystems, from low-lying areas to high mountains and from cold to tropical regions. Within this family, the genus *Artemisia* contains approximately 200–400 species (Mohamed et al. 2010). Numerous bioactive phytochemical compounds have been identified in *Artemisia*, including essential oils, sesquiterpene lactones, flavonoids, coumarins, artemisinin, arglabin, and other phenolics (Preedy 2016, Ivanescu et al. 2015, Crozier et al. 2006, Stefanachi et al. 2018, Cui and Su 2009, Mehdikhani et al. 2025, Clifford 2000, Zhang et al. 2026).

*Artemisia* plants are used in traditional medicines of many countries for a variety of indications, and they display a broad spectrum of biological activities, including insecticidal, anti-tubercular, antiparasitic, antimalarial, antiviral, and antimicrobial effects (Bisht et al. 2021, Suvaithenamudhan et al. 2022, Amin et al. 2019). *Artemisia abyssinica*, which grows in Saudi Arabia and other regions, is used as a decorative and therapeutic herb. The fresh aerial parts have been traditionally decocted and used for antibacterial, antileishmanial, anthelmintic, antirheumatic, antispasmodic, antitumor and antioxidant purposes (Youssef et al. 2015, Abad et al. 2012, Tesfaye et al. 2024, Rabey and Almutairi 2025).

The biological activities of *Artemisia* species are primarily attributed to their rich content of secondary metabolites, particularly phenolic compounds and flavonoids. Secondary metabolites are low-molecular-weight organic compounds synthesized by plants as defense mechanisms and stress responses (Trifan et al 2022, Shinyuy et al 2023). These compounds possess diverse pharmacological properties, including antioxidant, antimicrobial, anti-inflammatory, and anticancer activities (Trifan et al. 2022, Shinyuy et al. 2023, Ekiert et al. 2021). The specific composition and concentration of secondary metabolites vary among different *Artemisia* species and extraction methods, directly influencing their therapeutic efficacy. Therefore, comprehensive phytochemical characterization is essential to identify and quantify these bioactive compounds and establish structure-activity relationships that explain the traditional medicinal use of these plants (Trifan et al 2022, Shinyuy et al 2023).

Liquid chromatography–mass spectrometry (LC–MS/MS) and gas chromatography–mass spectrometry (GC–MS) are widely applied to characterize phytochemical constituents in natural-product extracts because of their sensitivity, selectivity, and accuracy (Stavrianidi 2019, Sparkman 2011]. For example, Trifan et al. (2022) examined methanol and chloroform extracts of five *Artemisia* species by LC–MS/MS and identified multiple coumarins, phenolic acids, sesquiterpene lactones, flavonoids, fatty acids, and a lignan (Trifan et al. 2022). Artemisinin was identified in several extracts of *Artemisia annua* using LC–MS/MS (Güzel et al. 2022), and quercetin was reported as an abundant antioxidant in *A. nilagirica* leaves by LC–MS/MS (Ivanescu et al. 2016).

The purpose of this study is to identify the chemical constituents of the methanolic extract of *Artemisia abyssinica* (MEAA) using LC–MS/MS and GC–MS analyses, and to evaluate its antioxidant activity, antimicrobial activity against selected bacterial strains, and exploratory cytotoxicity. Molecular docking studies were conducted to help rationalize observed bioactivities. Although the present investigation is experimental and in vitro in nature, the findings are intended to provide preliminary evidence supporting the identification of biologically active compounds with pharmaceutical relevance, as well as highlighting the potential of *Artemisia abyssinica* as a natural bioresource for future applications.

## Materials and methods

### Plant material

The aerial parts of the plant were obtained from a farm in Al-Mukwah region, Saudi Arabia in late January 2023. The roots were discarded. Plant identity was confirmed by Dr. Najeeb Alsagher, Assistant Professor of Forestry, Department of Biology, Faculty of Science, Al-Baha University. The specimens were stored at the Biology Department’s herbarium (No. AA 156-23), Faculty of Science, Al-Baha University, Saudi Arabia.

#### Extraction of the plant material

The fresh aerial parts of *A. abyssinica* were shade-dried at room temperature (25 ± 2 °C) for 7 days and subsequently ground into a fine powder using a mechanical grinder. A total of 100 g of powdered plant materials was extracted by maceration using methanol at a solid-to-solvent ratio of 1:5 (w/v). The extraction was performed in three successive cycles (3 × 500 mL), each for 72 h at room temperature (25 ± 2 °C) with occasional stirring to enhance solvent penetration.

After each extraction cycle, the mixture was filtered using Whatman No. 1 filter paper, and the combined filtrates were concentrated under reduced pressure at 40 °C using a rotary evaporator to remove the solvent. The resulting crude methanolic extract was further dried to obtain a viscous brown residue (6.5 g), designated as MEAA, and stored at 4 °C until further analysis.

#### LC-MS analysis

The MEAA was analysed by liquid chromatography tandem mass spectrometry (LC–MS/MS) in positive and negative ionization modes following previously described methods (Stavrianidi 2019, Sparkman 2011) with modifications. Chromatographic separation was performed on an ExionLC system (Sciex, USA) coupled to a TripleTOF 5600 + mass spectrometer (Sciex, USA). A detailed list of the instrumentation and software used in this study is provided in Table 1.

Chromatographic analysis was performed on a Phenomenex Kinetex^®^ C18 column (100 × 2.1 mm, 1.7 μm) maintained at 40 °C. The mobile phase consisted of solvent A (0.1% formic acid in water, v/v) and solvent B (0.1% formic acid in acetonitrile, v/v). Gradient elution was carried out as follows: 0–2 min, 5% B; 2–15 min, 5–40% B; 15–20 min, 40–95% B; 20–22 min, 95% B; 22–24 min, return to 5% B; and 24–27 min, 5% B for column equilibration. The flow rate was kept at 0.3 mL/min and the injection volume was 5 µL.

Detection was carried out using electrospray ionization (ESI) in both positive and negative ionisation modes. The mass scan range was m/z 100–1250. Other source parameters included ion spray voltage + 5500 V (positive) and − 4500 V (negative), source temperature 550 °C, curtain gas 35 psi, nebulizer gas 55 psi, heater gas 55 psi, and declustering potential 80 V.

**Table 1:** LC–MS/MS instrumentation and software used in this study

Item	Type	Vendor	Purpose	
ExionLC (High-flow LC)	Hardware	Sciex	Chromatographic separation	
TripleTOF 5600+	Hardware	Sciex	Mass spectrometric detection (IDA acquisition)	
Analyst TF 1.7.1	Software	Sciex	Instrument control and data acquisition	
PeakView	Software	Sciex	Data visualization and processing	
MS-DIAL	Software	RIKEN	Data processing and metabolite identification	

#### GC-MS analysis of MEAA

GC-MS analysis was performed without chemical derivatization to profile the volatile and semi-volatile constituents. Consequently, the identifications are considered reliable for compounds of appropriate volatility (e.g., certain terpenoids, fatty acids, alkanes). Pu-tative identifications for highly polar or glycosylated compounds made during automated database matching have been treated with extreme caution and, where implausible, removed from the final report. We used a Thermo Scientific Trace GC Ultra / ISQ Single Quadrupole MS with a TG-5MS fused silica capillary column (30 m, 0.25 mm, 0.10 μm film thickness) to perform the GC/MS analysis. The operating conditions were as previously described (Wang et al. 2018). Constituents were tentatively identified by comparing their mass spectra and relative retention times with those in the NIST and Wiley mass spectral libraries. It is important to note that Kovats retention indices were not calculated for this analysis due to the absence of a co-analyzed n-alkane standard series. Therefore, the reported identification results (Table 4), particularly for isomeric compounds, should be regarded as tentative based on spectral matching alone. The use of retention indices is highly recommended for more confident identification in future work.

### Biological screening

#### Screening of antimicrobial activity

The effect of MEAA on the growth of three Gram-positive bacteria (*Staphylococcus aureus* ATCC 25923, *Micrococcus luteus* clinical isolate and *Streptococcus pneumoniae* clinical isolate) and three Gram-negative bacteria (*Escherichia coli* ATCC 7839, *Escherichia coli* ATCC 25922 and *Salmonella typhi* ATCC 6539) was assessed using the well diffusion method (Balouiri et al. 2016). Ciprofloxacin antibiotic discs (5 µg) were used as a positive control and wells with 100 µL of DMSO were used as a negative control. Antibacterial activity was quantified by measuring the diameter (mm) of the inhibition zones surrounding the wells.

#### Minimum inhibitory concentration (MIC)

The MIC of MEAA using the broth macro-dilution method (Clinical and Laboratory Standards Institute 2018) was estimated against six bacterial strains. Test tubes were prepared with 2.5 mL of Tryptic Soy broth and MEAA dissolved in DMSO was added to achieve the desired concentrations. Based on the results of the initial agar well diffusion assay, more susceptible strains (those showing larger inhibition zones) were tested using a lower concentrations range (0.5, 1, 2, 4, 6, and 8 mg/mL) including *Micrococcus luteus*, *S. pneumoniae*, *E. coli* ATCC 7839 and *S. typhi* ATCC 6539, while concentrations of 1, 2, 4, 8, 10, and 12 mg/mL used for *S. aureus* ATCC 25,923 and E. coli ATCC 25,922. This approach was used to ensure the MIC for each strain would fall within its respective tested range. Each tube was inoculated with 10 µL of the corresponding bacterial suspension (adjusted to 0.1 O.D.) and mixed well. After incubation at 37 °C for 24 h, the MIC was recorded as the lowest concentration that completely inhibited the bacterial growth.

#### Minimum bactericidal concentration (MBC)

The MBC is the lowest concentration of the extract that kills 99.9% of the bacteria after 24 h of incubation. This procedure was performed by transferring a loop-full of the tubes with no bacterial growth from the MIC test to Tryptic Soy agar plates and incubating them at 37 °C for 24 h. The MBC/MIC ratio indicates if the extract is bactericidal or bacteriostatic against the bacteria. A ratio ≤ 4 means bactericidal and a ratio > 4 means bacteriostatic (Andrews 2001). The tests were repeated at least twice. Results are presented as the mean ± standard deviation.

### Antitumor activity

Hepatocellular carcinoma (HepG-2) cell line was obtained from the National Cancer Institute and cultured as a “monolayer” in RPMI medium with 10% FBS and 2% Pen/Strep. The cells were kept at 37 °C with 5% CO_2_ and high humidity in a water jacketed incubator (Thermo Fisher Scientific USA). We sub-cultured the cells regularly to maintain exponential growth. A laminar flow cabinet was used (Microflow Laminar flow cabinet, MDH Limited, Hampshire SP105AA, U.K.) to ensure sterile conditions. Cells were divided into a control group and treatment groups exposed to different concentrations of methanolic extract (12.5, 25, 50, and 100 µg/mL) (Skehan et al. 1990, Mosmann 1983, Denizot and Lang 1986).

#### Cell proliferation assay

To assess the cytotoxicity of the tested materials, MTT assay was performed as follows. After 24 h of incubation with the materials, 10 µL of MTT solution (0.5 mg/mL) was added to each well and the plate was incubated for another 4 h. Then, 100 µL of solubilization solution was added to dissolve the purple formazan crystals formed by viable cells. The absorbance of each well was measured at 570 nm using a microplate reader. The cell viability percentage was calculated using the formula: [ODS/ ODC] × 100, where ODS is the mean optical density of the sample and ODC is the mean optical density of the control. The results were presented as a graph of cell viability percentage versus material concentration using GraphPad Prism 8.0.2 software.

### Antioxidant activity

The stable radical 2,2-diphenyl-1-picrylhydrazyl (DPPH) was used to evaluate the antioxidant activity of MEAA by measuring its ability to donate hydrogen or scavenge radicals, following the described method (Carvalho et al. 2011). Different concentrations of the extract were tested, and the absorbance was measured spectrophotometrically at 517 nm. The percentage inhibition of DPPH radicals was calculated according to the standard formula. The IC50 was calculated from the concentration–response curve obtained by plotting the percentage of DPPH inhibition against the extract concentration. The linear regression analysis was applied to the experimental data obtained at the tested concentrations and the concentration required to obtain 50% radical inhibition (IC_50_) was calculated from the regression equation obtained.

### Molecular modeling

#### Small isolated molecule handling

Quantum mechanical calculations were employed to investigate key molecular characteristics, including electronic structure, physicochemical parameters, and excited-state behavior. The geometry of the target compounds was optimized using Density Functional Theory (DFT) and time-dependent DFT (TD-DFT) methods, specifically at the B3LYP/6–311G(d, p) level of theory.

#### Selection of protein structures

Molecular docking simulations were conducted to examine the binding interactions of the target compounds with the active sites of three proteins: 6M1J (antimicrobial target), 4HJO (antitumor target), and 4UBP (antioxidant target). The Molecular Operating Environment (MOE) 2015 software was used to prepare the protein structures. This involved correcting structural errors, optimizing geometry, resolving steric clashes, adding hydrogen atoms, and assigning partial charges using the Amber12:EHT forcefield. Energy minimization was subsequently carried out (AMBER12:EHT, RMS gradient: 0.100) to relax the structures prior to docking.

#### Analysis of binding site

The binding sites on the protein receptors were predicted using the Site Finder tool in MOE. This method utilizes a geometric approach based on alpha spheres—a generalization of convex hulls—to identify potential binding pockets without relying on energy-based models. The predicted binding sites corresponded to those occupied by co-crystallized ligands in the holo-structures of the proteins.

#### Docking procedure

The docking procedure began with the retrieval of the crystal structures of the target enzymes. Parameters and atomic charges were assigned using the MMFF94x forcefield. Alpha-site spheres within the binding site were generated using the Site Finder module. The optimized 3D structures of the ligands were docked using the triangular matcher placement method, which aligns triplets of ligand atoms with triplets of alpha spheres in the receptor site. Initial poses were rescored using the London dG scoring function, followed by refinement with the MMFF94x forcefield to account for solvation effects. The final binding energy of each pose was calculated using the GBVI/WSA dG solvation model and expressed in kcal/mol.

### Statistical analysis of data

All experimental analyses were performed in triplicate, and the results are expressed as mean ± standard deviation (SD). Statistical comparisons between experimental groups were performed using an independent *t*-test in Microsoft Excel for Microsoft 365 (Microsoft Corporation, Redmond, WA, USA). A *P* value < 0.05 was considered statistically significant.

## Results and discussion

### Investigation of the chemical constituents in methanolic extract

**Fig. 1:**
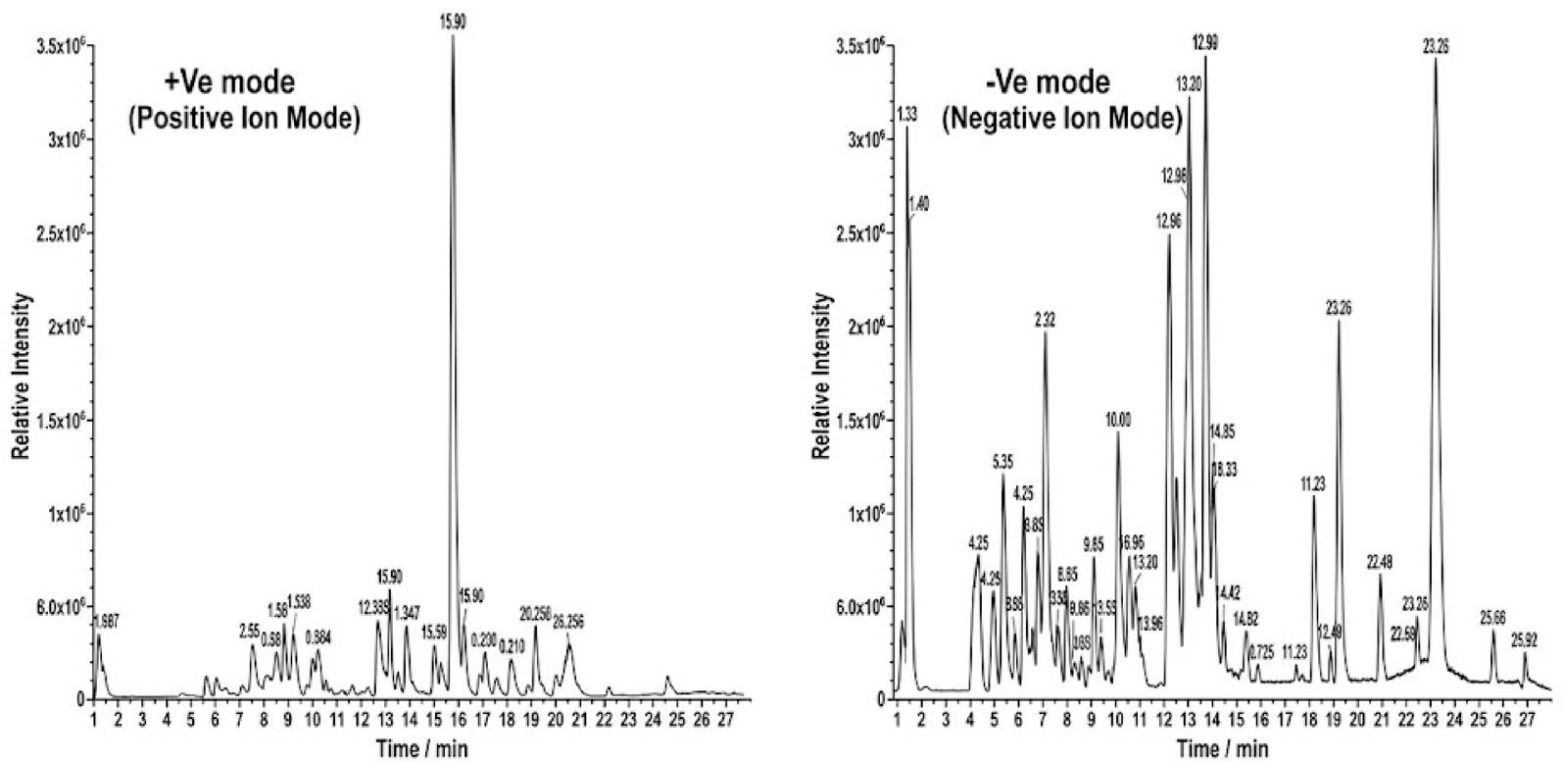
Liquid chromatography–mass spectrometry (LC–MS) chromatograms of the methanolic extract of *Artemisia abyssinica* (MEAA) acquired in positive and negative ionization modes

LC-MS analysis in both positive and negative ionization modes was used to characterize the chemical constituents of MEAA. Figure 1 presents the LC chromatogram obtained in the positive mode, while the identified compounds from both modes are summarized in Tables 2 and 3. The detected metabolites belong to four major chemical classes:

(i) Flavonoid aglycones and glycosides - including flavones, flavanones, flavonols, and flavanonols such as myricetin, acacetin, genkwanin, hispidulin, apigenin, apigenin-6-*C*-glucoside-7-*O*-glucoside, apigenin-7-*O*-glucoside, apigenin-7-*O*-hesperidoside, luteolin-4′-O-glucoside, hesperetin, quercetin, isorhamnetin, kaempferide, naringenin, taxifolin, and kaempferol-3-*O*-glucoside. These compounds exhibited characteristic protonated and deprotonated ions at m/z values such as 320, 285, 287, 299, 271, 595, 433, 577, 449, 301, 303, 317.
(ii) Cinnamaldehyde derivatives—including sinapaldehyde, coniferylaldehyde, and related compounds.
(iii) Anthocyanins—including anthocyanidin-3-O-glycosides, pelargonidin-3,5-di-*O-*glucoside, and delphinidin-3-*O*-glucoside.
(iv) *Coumarins*—such as scopoletin, 7-acetoxy-4-methyl coumarin, and 6,7-dihydroxycoumarin.

**Table 2:** Results of liquid chromatography–mass spectrometry (LC-MS) analysis in positive ionization mode showing tentatively identified compounds in the methanolic extract of *Artemisia abyssinica* (MEAA)

Peak no.	Retention time (min)	Relative abundance (%)	Molecular weight (m/z)	Molecular formula	Identified compounds
1	1.31	0.66	140	C_6_H_5_NO_3_	6-Hydroxynicotinic acid
2	4.68	0.15	176	C_10_H_9_NO_2_	1-methoxyindole-3-carbaldehyde
3	4.94	1.06	195	C_8_H_10_N_4_O_2_	Caffeine
4	5.29	0.57	410	C_24_H_40_O_5_	Cholic acid
5	5.46	0.31	341	C_15_H_16_O_9_	Esculin
6	6.23	10.06	208	C_11_H_12_O_4_	Sinapaldehyde*
7	6.47	10.05	320	C_15_H_10_O_8_	Myricetin*
8	7.08	1.76	449	C_21_H_20_O_11_	Kaempferol-3-*O*-glucoside
9	7.34	0.54	193	C_10_H_8_O_4_	Scopoletin
10	7.67	1.69	287	C_16_H_12_O_5_	Genkwanin
11	7.83	1.21	449	C_21_H_21_O_11_	Anthocyanidin-3-*O*-glycosides
12	7.91	1.32	433	C_21_H_20_O_10_	Apigenin-7-*O*-glucoside
13	8.01	1.29	179	C_10_H_10_O_3_	Coniferyl aldehyde
14	8.02	0.89	219	C_12_H_10_O_4_	7-Acetoxy-4-methylcoumarin
15	8.11	1.92	133	C_9_H_8_O	Cinnamaldehyde
16	8.21	1.20	431	C_22_H_22_O_9_	Ononin
17	9.74	6.56	303	C_15_H_10_O_7_	Quercetin
18	9.97	0.97	137	C_10_H_16_	Sabinene
19	10.21	5.82	273	C_15_H_12_O_5_	Naringenin
20	10.23	4.38	317	C_16_H_12_O_7_	Isorhamnetin
21	10.24	1.31	153	C_8_H_8_O_3_	Vanillin
22	10.29	0.26	165	C_9_H_8_O_3_	Hydroxycinnamic acids
23	10.32	7.51	301	C_16_H_12_O_6_	Kaempferide
24	10.56	0.28	229	C_14_H_12_O_3_	Resveratrol
25	10.66	10.71	305	C_15_H_12_O_7_	Taxifolin*
26	11.29	1.55	291	C_15_H_14_O_6_	Epicatechin
27	12.10	4.49	377	C_17_H_20_N_4_O_6_	Riboflavin
28	12.20	2.45	271	C_15_H_10_O_5_	Apigenin
29	14.10	11.79	285	C_16_H_12_O_5_	Acacetin*
30	17.66	1.69	301	C_20_H_28_O_2_	Retinoic acid
31	18.32	0. 91	463	C_21_H_18_O_12_	Kaempferol-3-Glucuronide
32	20.54	1.32	595	C_27_H_30_O_15_	Apigenin-6-*C*-glucoside − 7-*O-*glucoside
33	24.57	0.54	595	C_27_H_31_O_15_	pelargonidin-3,5-di-*O*-glucoside

*Compounds with relative abundance ≥ 10% are considered major constituents

These findings are consistent with previous reports describing the phytochemical composition and biological activities of *Artemisia* species using LC–MS-based analysis (Trifan et al. 2022, Mohammed et al. 2022).

Similar studies have reported the presence of several phenolic acids and flavonoids, including chlorogenic acid, quercetin derivatives, and caffeoylquinic acid derivatives, which are known for their antioxidant, anti-inflammatory, chemopreventive, and immunomodulatory properties. Likewise, the phytochemical profile of *A. vulgaris* has been reported to contain flavonoids derivatives of kaempferol and quercetin, coumarins (coumarin, esculin, scopoletin, and umbelliferone), phenolic acids (such as caffeic and chlorogenic acids), sterols, and carotenoids (Avula et al. 2009).

*Artemisia annua* has also been reported to produce phenylpropanoid glycosides (Abate et al. 2021). The similarity between these reported constituents and the major compounds detected in MEAA further supports the conserved secondary-metabolite biosynthesis across the *Artemisia* genus.

**Fig. 2:**
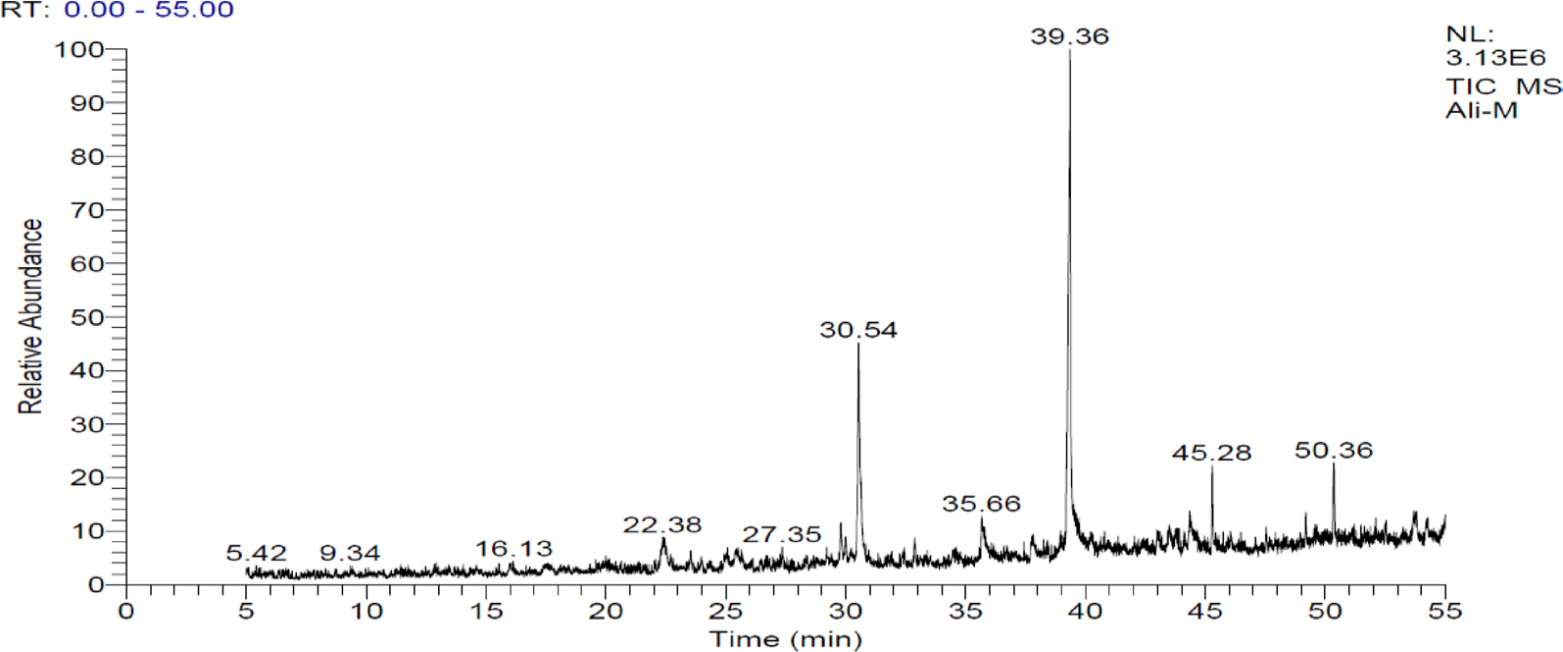
Gas chromatography (GC) chromatogram of the methanolic extract of *Artemisia abyssinica* showing tentatively identified compounds (MEAA)

GC–MS analysis, suitable for profiling volatile and semi-volatile constituents, tentatively identified 19 compounds (Table 4; Fig. 2), with gitoxigenin (28.55%) and diplodialide-B (16.58%) as the most abundant constituents. Other detected metabolites included fucoxanthin (3.02%), *n*-nonacosane (6.45%), and palmitic acid (3.34%), representing typical lipophilic constituents such as terpenoids, fatty acids, hydrocarbons, and carotenoid derivatives. Similar classes of compounds have been reported in *Artemisia* species analyzed by GC–MS (Aati et al. 2021, Belyagoubi-Benhammou et al. 2024).

**Scheme 1:**
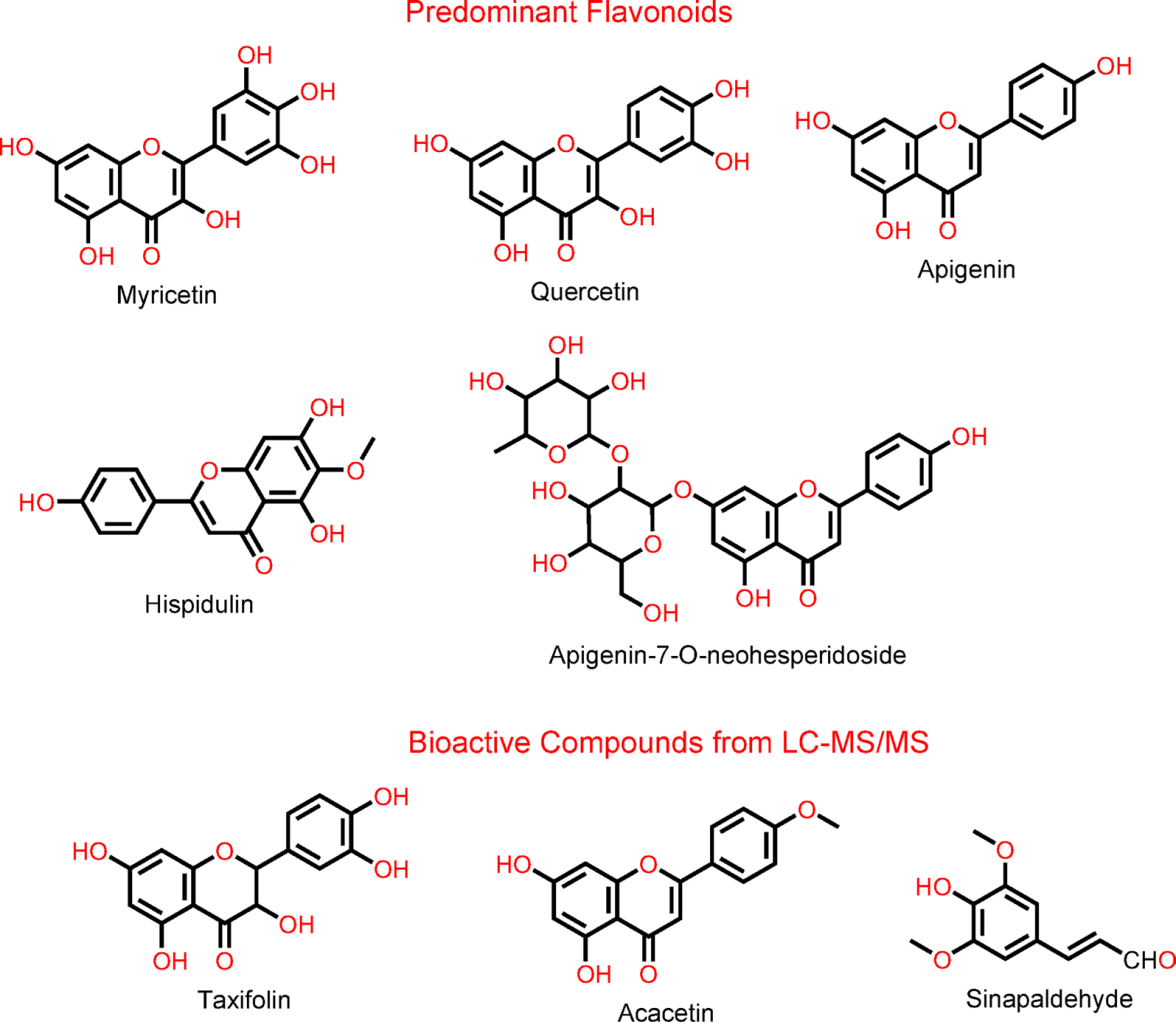
Chemical structures of selected phytochemical compounds identified in the methanolic extract of *Artemisia abyssinica* (MEAA) based on LC–MS/MS and GC–MS analyses

It is important to emphasize that GC–MS is inherently limited to volatile and semi-volatile molecules; therefore, polar and non-volatile metabolites, including glycosylated flavonoids, are not detectable without chemical derivatization. Accordingly, initial automated matches suggesting diglycosylated flavonoids were considered analytical artifacts and were removed from the final GC–MS profile. The GC–MS results should therefore be interpreted strictly as representing the volatile fraction of MEAA and as complementary to LC–MS/MS, which remains the primary technique for profiling polar phytochemicals. These findings support the chemical richness of MEAA and its biological relevance (Shinyuy et al 2023, Ekiert et al 2021, Belyagoubi-Benhammou et al 2024). This phytochemical diversity further highlights the potential of *A. abyssinica* as a valuable bioresource for the discovery of functionally relevant natural compounds (Scheme 1).

**Table 3:** Results of liquid chromatography–mass spectrometry (LC-MS) analysis in negative ionization mode showing tentatively identified compounds in the methanolic extract of *Artemisia abyssinica* (MEAA)

Peak no.	Retention time (min)	Relative abundance %	Molecular weight (m/z)	Molecular formula	Identified compounds
1	1.22	0.47	153	C_7_H_6_O_4_	4-Hydroxybenzoic acid
2	1.32	1.70	151	C_5_H_4_N_4_O_2_	Xanthine
3	1.35	0.58	353	C_16_H_18_O_9_	Chlorogenic Acid
4	1.39	2.1	163	C_9_H_8_O_3_	Hydroxycinnamic acid
5	1.46	1.78	133	C_6_H_12_O_3_	2-Hydroxy-4-methylpentanoate
6	1.62	2.15	283	C_16_H_12_O_5_	Acacetin
7	1.94	1.00	165	C_9_H_10_O_3_	Phenyllactic acid
8	6.12	0.04	431	C_21_H_20_O_10_	Apigenin 8-*C*-glucoside
9	6.18	0.06	447	C_21_H_21_O_11_	Cyanidin-3-*O*-galactoside
10	6.45	2,92	385	C_17_H_22_O_10_	1-OD-glucopyranosyl sinapate
11	6.66	0.16	463	C_21_H_21_O_12_	Delphinidin-3-*O*-glucoside
12	6.88	0.17	191	C_10_H_8_O_4_	Scopoletin
13	6.93	0.09	623	C_28_H_32_O_16_	Isorhamnetin-3-*O*-rutinoside
14	7.21	0.52	477	C_22_H_22_O_12_	Isorhamnetin-3-*O*-glucoside
15	7.26	1.04	447	C_21_H_20_O_11_	Luteolin-4’-*O*-glucoside
16	7.37	20.43	269	C_15_H_10_O_5_	Apigenin*
17	7.39	5.06	431	C_21_H_21_O_10_	pelargonidin-3-*O*-glucoside
18	7.42	8.26	577	C_27_H_30_O_14_	Apigenin 7-*O*-neohesperidoside
19	7.90	0.49	463	C_21_H_20_O_12_	Hyperoside (Quercetin 3-galactoside)
20	7.96	2.27	151	C_8_H_8_O_3_	2(hydroxyphenyl)acetic acids
21	7.99	0.94	179	C_9_H_8_O_4_	Caffeic acid
22	8.39	1.64	405	C_20_H_22_O_9_	Astringin
23	8.75	0.60	177	C_9_H_6_O_4_	6,7-dihydroxycoumarin
24	8.98	0.42	417	C_20_H_18_O_10_	Kaempferol-3-*O* -arabinoside
25	9.12	1.94	287	C_15_H_12_O_6_	naringenin
26	10.24	5.17	301	C_16_H_14_O_6_	Hesperetin
27	10.93	36.78	299	C_16_H_12_O_6_	Hispidulin*
28	11.37	0.85	174	C_6_H_13_N_3_O_3_	Citrulline
29	20.97	0.19	289	C_15_H_14_O_6_	Catechin

*Compounds with relative abundance ≥ 10% are considered major constituents

**Table 4:** Selected phytochemical compounds identified in the methanolic extract of *Artemisia abyssinica* (MEAA) based on LC–MS analysis

Peak no.	Retention time (min)	Relative abundance (%)	Mass data			Identified compound
			Molecular weight	Base peak (m/z)	Molecular formula	
1	3.16	1.60	228	58	C_13_H_24_O_3_	12-oxo-tridecanoic acid
2	5.72	2.50	212	169	C_11_H_16_O_4_	5-Isopropyl7,6-dioxabicycl [3.2.1]octane-2-oic acid
3	29.79	2.94	554	69	C_40_H_58_O	Rhodopin
4	30.54	16.58	184	70	C_10_H_16_O_3_	Diplodialide-B*
5	32.88	2.35	240	43	C_15_H_28_O_2_	E-8-Methyl-7-dodecen-1-ol acetate
6	35.67	3.34	256	43	C_16_H_32_O2	Palmitic acid
7	37.76	1.26	436	43	C_26_H_44_O_5_	Ethyl isoallocholate
8	39.36	28.55	390	203	C_23_H_34_O_5_	Gitoxigenin*
9	40.25	1.10	536	91	C_40_H_56_	Carotene
10	40.97	1.25	426	109	C_27_H_38_O_4_	Azafrin
11	43.02	2.12	314	85	C_19_H_38_O_3_	Methyl 4-hydroxyoctadecanoate
12	44.36	3.02	658	44	C_42_H_58_O_6_	Fucoxanthin
13	45.29	6.45	408	43	C_29_H_60_	*n*-Nonacosane
14	51.14	1.43	596	91	C_40_H_52_O_4_	Astaxanthin
15	51.63	0.83	450	57	C_32_H_66_	n-Dotriacontane
16	52.50	1.56	458	43	C_29_H_46_O_4_	Viperidone acetate

*Compounds with relative abundance ≥ 10% are considered major constituents

### Antimicrobial activity

The investigated MEAA was screened for its activity against Gram-negative and Gram-positive bacteria. Agar well diffusion results (Table 5) revealed that it exhibited moderate antimicrobial activity against both Gram-negative and Gram-positive bacteria. However, *S. aureus* and *E. coli* (ATCC 25922) strains were resistant to the extract at the tested concentration (5 mg/mL). The absence of inhibition zones against *S. aureus* and *E. coli* in the agar diffusion assay at 5 mg/mL does not necessarily indicate complete inactivity, as these strains exhibited inhibitory responses at higher concentrations during the broth macrodilution MIC assay. This difference may reflect the limited diffusion of certain phytochemical constituents in agar media compared with liquid broth conditions.

The differential susceptibility observed between *E. coli* ATCC 7839 (sensitive, inhibition zone 11 mm) and *E. coli* ATCC 25,922 (resistant, no inhibition zone) warrants discussion. This variation may be attributed to strain-specific differences such as outer membrane permeability, efflux pump expression, or lipopolysaccharide structure (Rivera et al. 1993). ATCC 25,922 is a standard quality control strain known to have a well-regulated efflux system and compact lipopolysaccharide layer, which may limit the penetration of certain phytochemical constituents (Rivera et al. 1993). In contrast, ATCC 7839 may possess structural differences in its outer membrane that facilitate greater uptake of phenolic compounds. Strain-dependent susceptibility patterns may explain the observed variations.

**Table 5:** Antibacterial activity of the methanolic extract of *Artemisia abyssinica* (MEAA) at a concentration of 5 mg/mL

Sample	Inhibition zone diameter (mm)					
	Gram-Positive			Gram-Negative		
	S. pneumoniae	M. luteus	S. aureus	E. coli*^1^	E. coli*^2^	S. typhi
Methanolic extract	11 ± 0.5	15 ± 1	ND	11 ± 0.5	ND	12.5 ± 0.5^#^
Negative control (DMSO)	ND	ND	ND	ND	ND	ND
Ciprofloxacin (5 µg)	35 ± 2	34 ± 1	34 ± 1	35 ± 1	40 ± 2	38 ± 1

*S. pneumoniae* = *Streptococcus pneumoniae*; *M. luteus* = *Micrococcus luteus*; *S. aureus* = *Staphylococcus aureus* (ATCC 25923); *E. coli* = *Escherichia coli*; *S. typhi* = *Salmonella typhi* (ATCC 6539); *^1^ = ATCC 7839; *^2^ = ATCC 25,922; DMSO= Dimethyl sulfoxide; ^#^ = reduced susceptibility. Data are expressed as mean ± standard deviation (SD) calculated from three replicates. ND stands for no zone of inhibition observed

**Fig. 3:**
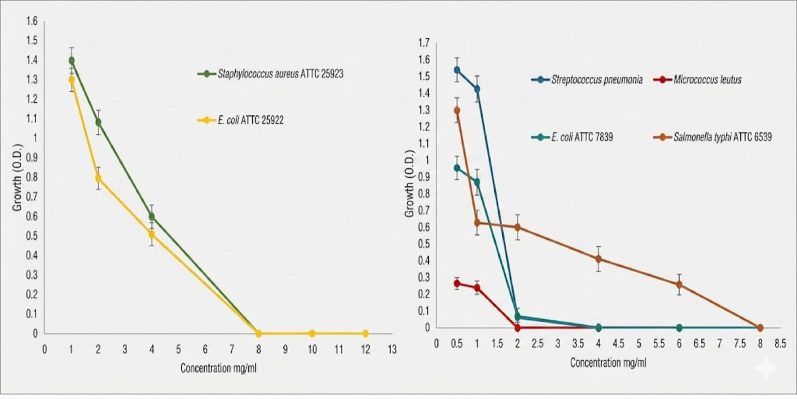
Concentration-response curve of the methanolic extract of *Artemisia abyssinica* (MEAA) against selected Gram-positive and Gram-negative bacterial strains. Growth is expressed as optical density (OD). Error bars indicate the standard deviation from three replicates

The Minimum Inhibitory Concentration (MIC) of MEAA was determined using the broth macrodilution technique. The extract showed its lowest MIC value against *M. luteus*, with complete inhibition at 2 mg/mL, whereas a concentration of 4 mg/mL was required to inhibit *S. pneumoniae* and *E. coli* (ATCC 7839).The antimicrobial effect against these organisms was bactericidal, as confirmed by the absence of visible growth upon sub-culturing. In contrast, the remaining tested bacteria were inhibited only at higher concentration (8 mg/mL), as illustrated in Table 6; Fig. 3. The antibacterial activity of MEAA is likely attributed to its phenolic and flavonoid constituents, as previously described (Shinyuy et al 2023, Ekiert et al 2021, Belyagoubi-Benhammou et al 2024).

The antimicrobial activity recorded for MEAA aligns with previously reported findings for other *Artemisia* species. Methanolic extract of *A. dracunculus* exhibited notable activity against *Shigella* species, *Listeria monocytogenes*, *Pseudomonas aeruginosa* and *E. coli*, supporting the role of phenolics and flavonoids in mediating antibacterial effect (Shinyuy et al. 2023). Similarly, inhibition zones of 11–14 mm for methanolic extract of *A. vulgaris*, which they attributed to the high levels of polyphenolic constituents (Ekiert et al. 2021). The current data for MEAA (11–15 mm against *S. pneumoniae*, *M. luteus*, and *E. coli* ATCC 7839) fall within this previously reported range, reinforcing the idea that methanolic extracts rich in flavonoids display moderate-to-strong antibacterial activity. Methanolic extracts of *A. absinthum*, and *A. santonicum* were more active (6–18 mm and 6–19 mm sizes for inhibition zones, respectively) than aqueous extracts, again emphasizing the extraction solvent’s influence on bioactivity (Aati et al. 2021). In addition, ethanolic extracts of *A. absinthium* and *A. annua* showed inhibitory effects against *S. aureus*, *E. coli*, *S. enteritidis*, and *L. monocytogenes* (Bordean et al. 2023), which aligns with the MIC values observed for MEAA in the present investigation. Overall, these comparisons indicate that the antibacterial efficacy of MEAA is comparable to that of other well-studied *Artemisia* species and is likely driven by its abundant flavonoids and phenolic acids.

**Table 6:** Minimum inhibitory concentration (MIC), minimum bactericidal concentration (MBC) and MBC/MIC ratio of the methanolic extract of *Artemisia abyssinica* (MEAA)

Methanolic extract (MEAA)	Gram-positive			Gram-negative		
	S. pneumoniae	M. luteus	S. aureus	E. coli*^1^	E. coli*^2^	S. typhi
MIC (mg/mL)	4.0	2.0	8.0	4.0	8.0	8.0
MBC (mg/mL)	4.0	2.0	> 12	4.0	> 12	> 8
MBC/MIC ratio (interpretation)	1 bactericidal	1 bactericidal	ND	1 bactericidal	ND	ND

*S. pneumoniae* = *Streptococcus pneumoniae*; *M. luteus* = *Micrococcus luteus*; *S. aureus* = *Staphylococcus aureus* (ATCC 25923); *E. coli* = *Escherichia coli*; *S. typhi* = *Salmonella typhi* (ATCC 6539); *^1^= ATCC 7839; *^2^= ATCC 25,922; ND: Not detected within the tested concentration range.MBC/MIC ≤ 4 indicates bactericidal activity; MBC/MIC > 4 indicates bacteriostatic activity

### Antioxidant activity

MEAA exhibited weak antioxidant activity, with a DPPH IC_50_ value of 268.15 µg/mL (Table 7). However, Thangjam et al. (Becke 1993), showed that methanolic extracts of *A. vulgaris* exhibited comparable DPPH scavenging activity, with stronger effects observed in samples containing higher levels of flavonoids and phenolic acid. Likewise, extracts of *A. campestris* and *A. hirsuta* display antioxidant activities closely correlated with phenolic abundance (Lee et al. 1988). Taken together, these studies support the present results, indicating that the flavonoids and phenolic acids identified in MEAA likely contribute to its observed antioxidant properties.

**Table 7:** Antioxidant activity of the methanolic extract of *Artemisia abyssinica* (MEAA) determined using the DPPH radical-scavenging assay

Tested extract	DPPH % inhibition				
Concentration (µg/mL)	100	50	25	12	IC_50_ (µg/ mL)
Methanol	24.43 ± 0.66	15.13 ± 0.01	12.08 ± 0.57	11.10 ± 0.57	268.15*
Ascorbic acid	95.98 ± 0.36	82.46 ± 0.79	52.11 ± 0.19	20.83 ± 2.50	25.19

Values are expressed as mean ± SD (*n* = 3). IC_50_ = concentration required to inhibit 50% of DPPH radicals. * indicates a statistically significant difference compared to the ascorbic acid reference control group (*P* < 0.001) using an independent *t*-test

### Cytotoxicity study

**Fig. 4:**
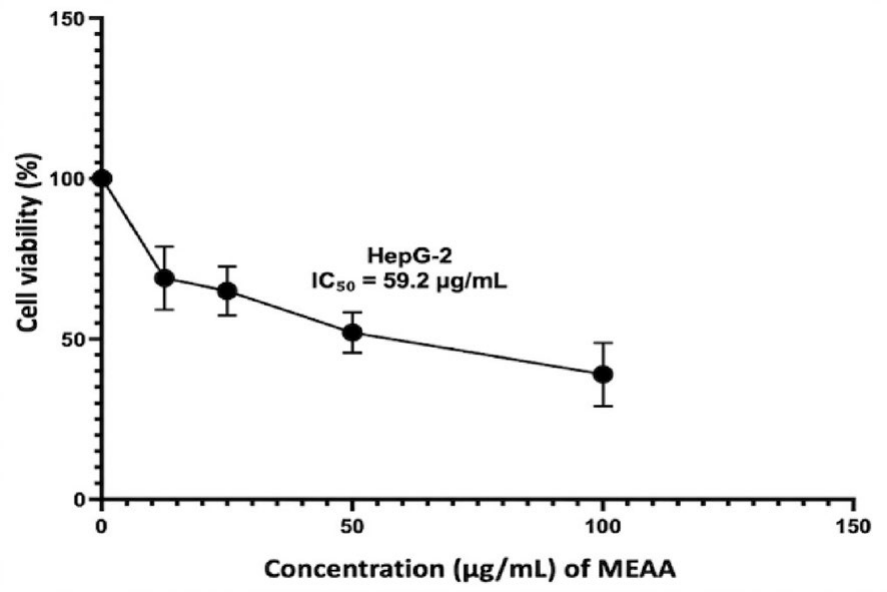
Dose–response curve of the methanolic extract of *Artemisia abyssinica* (MEAA) against hepatocellular carcinoma cells (HepG-2) determined by cytotoxicity assay. The IC_50_ value was calculated as 59.2 µg/mL. Error bars indicate the standard deviation

The cytotoxic activity of MEAA against hepatocellular carcinoma (HepG-2) cells showed a moderate effect, with an IC_50_ value of 59.2 µg/mL (Fig. 4), compared with the reference drug Adriamycin (IC_50_ = 6.9 µg/mL). Similar moderate cytotoxic effects have been documented for other *Artemisia* species rich in phenolic constituents. Jakovljević et al. (Radović Jakovljević et al 2023, Kim and Choi 2013) reported that flavonoids, phenolic acids, and selected terpenoids isolated from *Artemisia* extracts contribute substantially to antiproliferative activity. In addition, extract of *Artemisia princeps* var. *orientalis* inhibit the growth of HepG-2 and Hep3B liver cancer cell lines (Mohamed et al 2025, Qanash et al 2023). These findings demonstrate that the phenolic and terpenoidal constituents detected in MEAA are consistent with the cytotoxic effects reported for related *Artemisia* species. It should be noted that the cytotoxicity evaluation in the present study was limited to cancer cell lines, and no normal hepatocyte cell lines were included for comparison. Therefore, the results should be considered a preliminary assessment of antiproliferative activity. Further studies incorporating normal cell lines are required to evaluate the selectivity and safety profile of the extract.

### Molecular docking studies

Molecular docking simulations were performed to evaluate the potential interactions of Sinapaldehyde, Taxifolin, Acacetin, Myricetin, Apigenin, and Hispidulin with the active sites of DNA gyrase (PDB: 6M1J), EGFR tyrosine kinase (PDB: 4HJO), and urease (PDB: 4UBP). These compounds were selected based on the following criteria; (i) they represent the major phytochemical classes detected in MEAA by LC–MS/MS, including flavonoids (myricetin, taxifolin, acacetin, apigenin, hispidulin) and a phenylpropanoid derivative (sinapaldehyde); (ii) they were among the most abundant compounds identified in the extract, with relative abundances exceeding 5% in the LC–MS/MS profile; (iii) flavonoids of these structural types have been previously reported to exhibit antimicrobial, antioxidant, and anticancer activities in related *Artemisia* species; and (iv) these compounds possess diverse substitution patterns (hydroxylation, methoxylation, glycosylation) that allow for a comparative SAR analysis across the three selected protein targets.

The docking protocol was validated by re-docking the co-crystallized ligands into their respective binding sites. For DNA gyrase (PDB: 6M1J), the co-crystallized inhibitor resulted in an RMSD of 0.82 Å (ΔG = − 8.45 kcal/mol). For EGFR (PDB: 4HJO), erlotinib (co-crystallized ligand) achieved an RMSD of 0.91 Å (ΔG = − 9.23 kcal/mol). For urease (PDB: 4UBP), acetohydroxamic acid (co-crystallized inhibitor) gave an RMSD of 0.76 Å (ΔG = − 5.89 kcal/mol). These low RMSD values (< 1.5 Å) confirm the reliability of the docking procedure and parameter settings (Qanash et al. 2023). These computational analyses aimed to provide structural insight into how these compounds may bind to the selected targets. The docking simulations were performed using the Glide module.

The binding free energies (ΔG) obtained for each compound-protein complex are presented in Table 8. The docking poses showed that all evaluated molecules were able to occupy the active sites of the three proteins and establish interactions with key amino acid residues. These interactions were then used to compare the predicted binding strengths of the identified compounds.

#### Binding efficacy

Docking simulations using the MOE platform (Chemical Computing Group ULC 2015) provided additional details regarding the interaction profiles of the identified compounds. According to the calculated ΔG values, Myricetin, Apigenin, and Hispidulin displayed more favorable predicted binding affinities compared with Sinapaldehyde, Taxifolin, and Acacetin. These differences are reflected in the interaction energies summarized in Table 8. It is important to note that these docking predictions describe theoretical binding preferences and do not directly represent biological potency; however, they offer supportive structural information that complements the experimental findings.

In agreement with previously published docking investigations on *Artemisia*-derived flavonoids, the present docking scores and interaction patterns demonstrate similar trends in binding preference. Several studies have reported that flavonoids such as myricetin, apigenin, and hispidulin consistently exhibit relatively high predicted binding affinity toward DNA gyrase, EGFR, and urease through hydrogen-bonding with catalytic residues and π–π stacking within the binding pocket. Comparable binding energies have been documented for Artemisia extracts evaluated against microbial and cancer-related targets (Suvaithenamudhan et al. 2022, Kim and Choi 2013, Mohamed et al. 2025, Qanash et al. 2023), where flavonoid-rich fractions showed enhanced stability within gyrase and EGFR active sites. Since the compounds were not isolated and tested individually, the docking results are presented as preliminary and hypothesis-generating, rather than definitive evidence of biological activity. The predicted interactions highlight potential contributors within MEAA, but the biological relevance of each compound requires further experimental validation. Furthermore, if any compound assignments from the GC–MS analysis are revised, the relevance of the docking results may need to be reconsidered.

**Table 8:** Docking free-energy scores (ΔG, Kcal/mol) and RMSD values of isolated components docked against DNA gyrase (6M1J), EGFR (4HJO), and urease (4UBP)

Compound	6M1J		4HJO		4UBP	
	ΔG	RMSD	ΔG	RMSD	ΔG	RMSD
Sinapaldehyde	− 5.963	1.405	− 5.721	1.486	− 5.100	1.673
Taxifolin	− 5.945	1.607	− 6.456	0.933	− 6.616	0.928
Acacetin	− 5.904	0.887	− 6.609	1.147	− 5.716	3.140
Myricetin	− 6.676	1.230	− 6.734	1.147	− 6.620	2.072
Apigenin	− 6.150	1.308	− 6.417	2.613	− 6.221	0.717
Hispidulin	− 6.817	0.848	− 6.447	1.291	− 5.322	3.391
erlotinib	–	–	− 7.365	0.625	–	–
Acetohydroxamic Acid	–	–	–	–	7.360	1.365
Ciprofloxacin	8.260	1.123	–	–	–	–

ΔG values represent predicted binding free energy (kcal/mol). RMSD indicates root-mean-square deviation between redocked and crystallographic poses. Lower ΔG values indicate relatively higher predicted binding affinity

These parallels reinforce the current findings that the MEAA flavonoids identified by LC–MS/MS represent structurally suitable ligands capable of forming energetically favorable and functionally relevant interactions with all three targets

#### Chemical interaction with 6M1J domain as antimicrobial

**Fig. 5:**
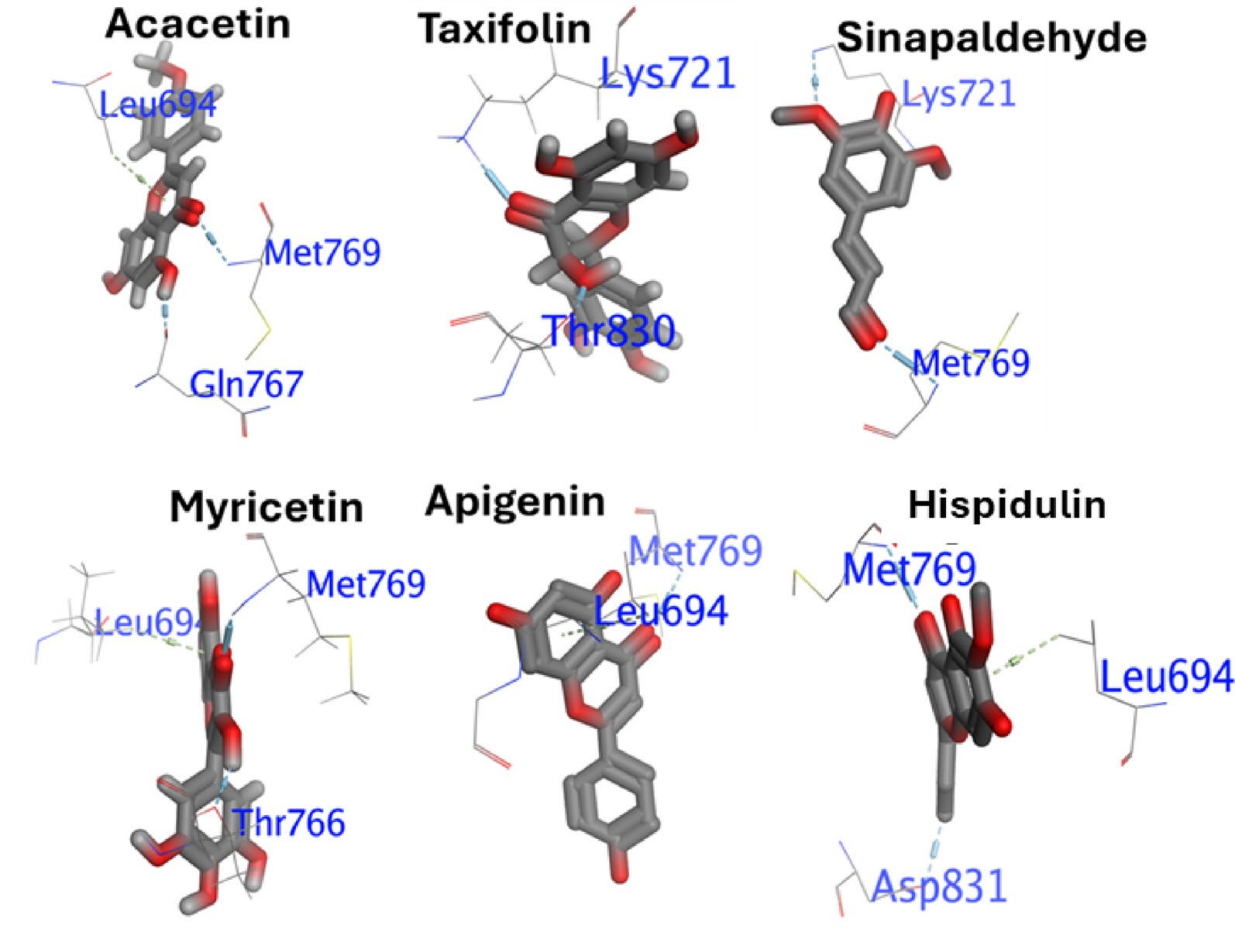
Three-dimensional binding conformations of Sinapaldehyde, Taxifolin, Acacetin, Myricetin, Apigenin, and Hispidulin within the active DNA gyrase (PDB: 6M1J), highlighting key amino acid residues involved in ligand stabilization. Green dashed lines represent hydrogen bonds; orange surfaces indicate hydrophobic regions; purple dashed lines denote π-π stacking interactions. Key amino acid residues involved in ligand stabilization are labelled

To visualize ligand–protein interactions, three-dimensional docking poses were analyzed as shown in Fig. 5. All tested compounds occupied the active-site cavity of DNA gyrase (PDB: 6M1J) with stable binding orientations. Hydrogen-bonding interactions with catalytically relevant residues, including Glu52 and Asp75, were observed for several ligands, suggesting favorable polar contacts within the enzymatic pocket.

Hydrophobic surface analysis further revealed that non-polar regions of the binding site contribute to ligand stabilization through van der Waals interactions, particularly for Gitoxigenin, Diplodialide-B, Fucoxanthin and *n*-Nonacosane. Blue regions in Fig. 5 indicate hydrophobic zones, whereas red regions represent hydrophilic surfaces. Collectively, these interaction patterns provide a structural explanation for the predicted binding behavior of the tested compounds against DNA gyrase and support their potential contribution to antimicrobial activity at the molecular level.

#### Chemical interaction with 4 HJO domain as antitumor

**Fig. 6:**
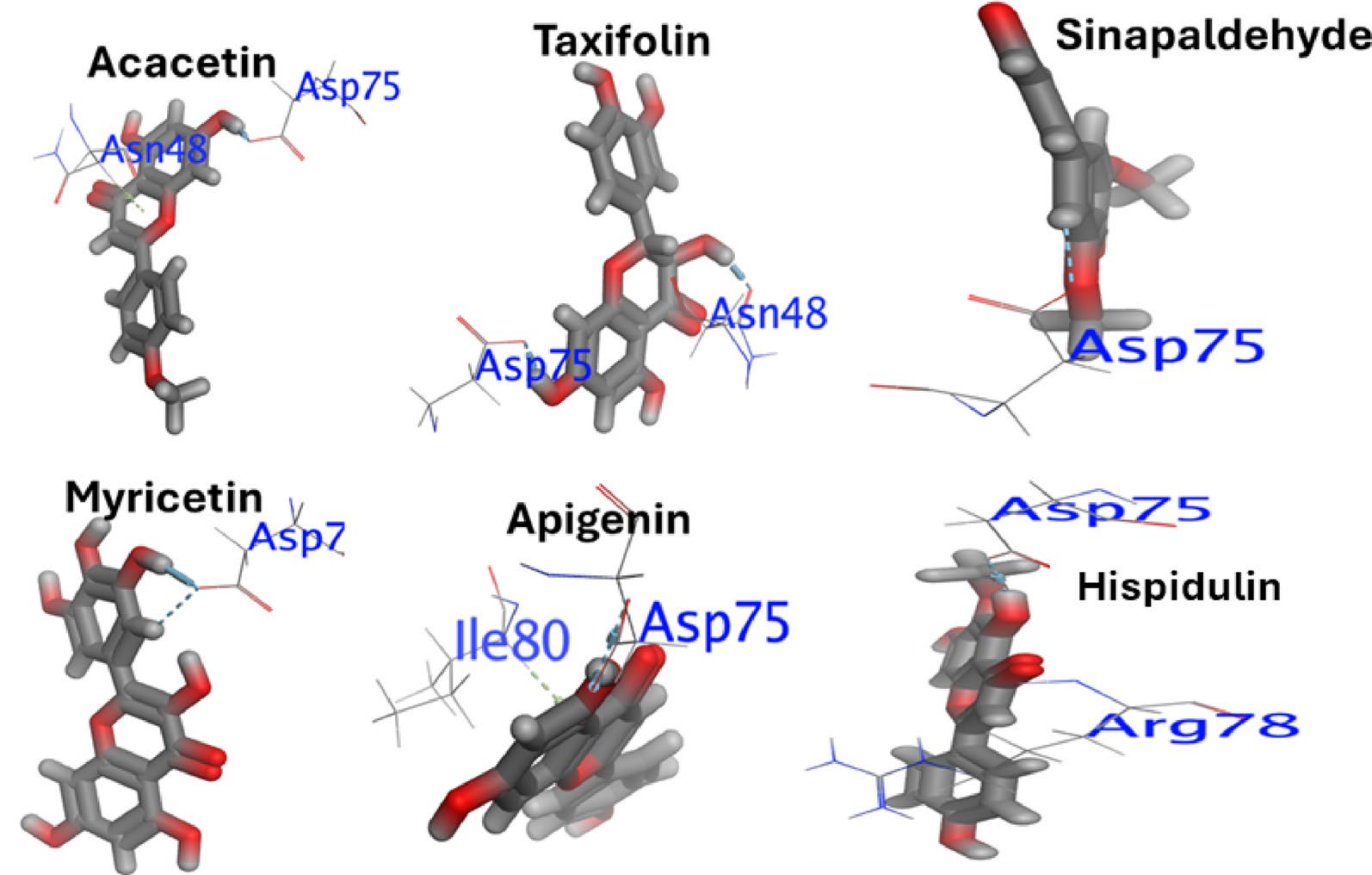
Three-dimensional binding conformations of Sinapaldehyde, Taxifolin, Acacetin, Myricetin, Apigenin, and *Hispidulin* within the active site of EGFR tyrosine kinase (PDB: 4HJO), showing key hydrogen-bonding and hydrophobic interactions. Green dashed lines represent hydrogen bonds; orange surfaces indicate hydrophobic regions; purple dashed lines denote π-π stacking interactions. Key amino acid residues involved in ligand stabilization are labelled

Binding analysis against EGFR tyrosine kinase (PDB: 4HJO) revealed that Acacetin, Myricetin, Apigenin, and Hispidulin established hydrogen- bond interactions with key residues within the ATP-binding site, including Met769, as illustrated in Fig. 6. These molecular interactions indicate favorable alignment within the catalytic pocket and suggest potential interference with receptor signaling based on predicted structural compatibility.

#### Chemical interaction with 4UBP domain as antioxidant

**Fig. 7:**
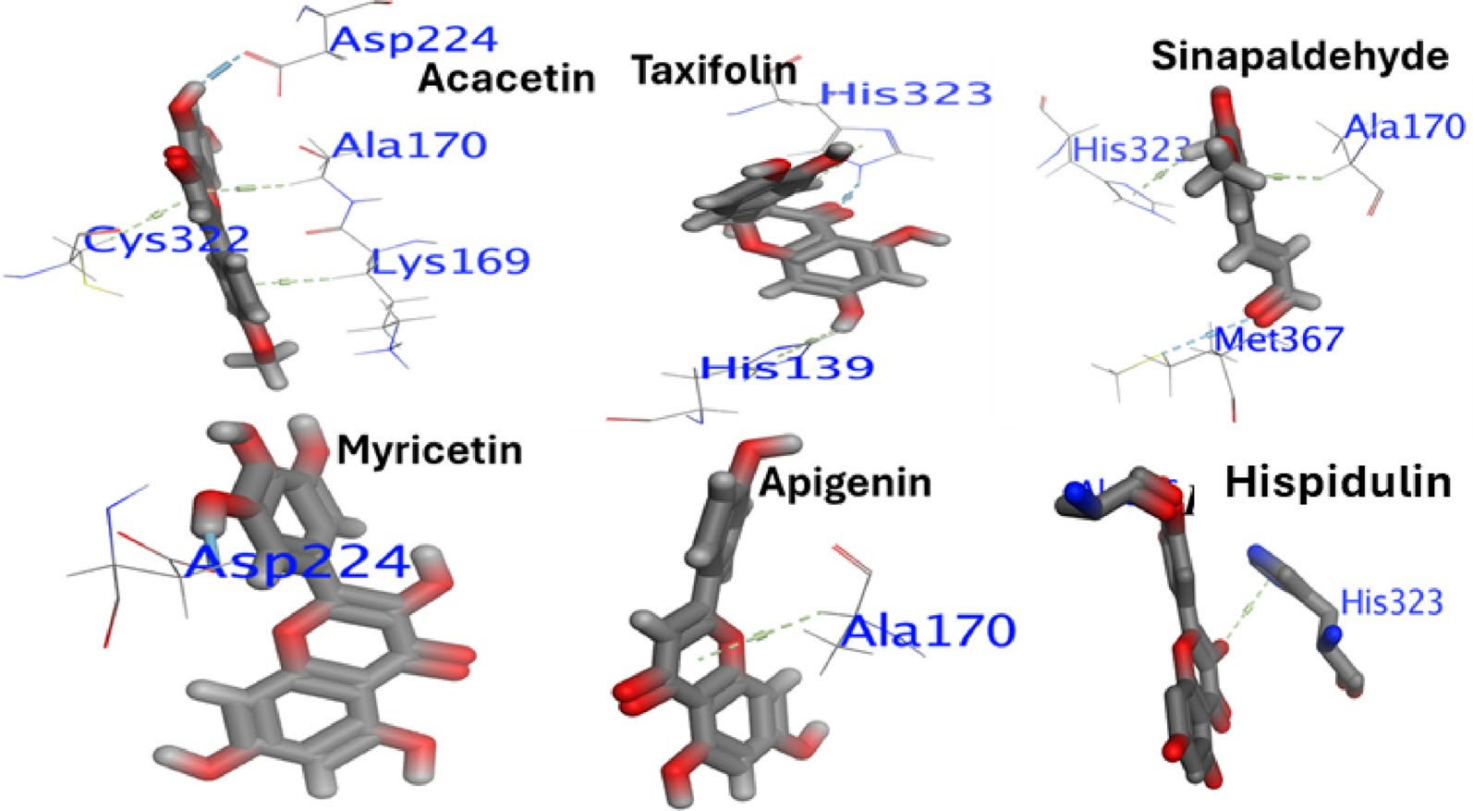
Three-dimensional binding conformations of Sinapaldehyde, Taxifolin, Acacetin, Myricetin, Apigenin, and Hispidulin within the active site of urease (PDB: 4UBP). Green dashed lines represent hydrogen bonds; orange surfaces indicate hydrophobic regions; purple dashed lines denote π-π stacking interactions. Key amino acid residues involved in ligand stabilization are labelled

Molecular docking against urease (PDB: 4UBP) suggested that Acacetin, Myricetin, Apigenin, and Hispidulin interact favorably within the enzyme’s active site through hydrogen-bonding and polar contacts with residues involved in substrate recognition (Fig. 7). These binding features support the proposed antioxidant-related enzyme targeting observed in silico.

In agreement with previously published investigations on *Artemisia* extracts, the present findings demonstrate a consistent pattern across phytochemical, antimicrobial, antioxidant, cytotoxic, and in silico evaluations. Earlier studies have reported that flavonoid- and phenolic-rich *Artemisia* species exhibit moderate antimicrobial and limited antioxidant effects, together with measurable cytotoxic activity against hepatocellular carcinoma cells (Suvaithenamudhan et al. 2022, Shinyuy et al. 2023, Mohammed et al. 2022, Lee et al. 1988, Chemical Computing Group ULC 2015, Jakovljević et al. 2020, Radović Jakovljević et al. 2023, Kim and Choi 2013). The phytochemical profiles identified in MEAA, particularly the presence of myricetin, apigenin, hispidulin, taxifolin, and phenolic acids, correspond closely with those reported for *A. argyi*, *A. vulgaris*, and *A. campestris*, supporting conserved biosynthetic patterns within the genus. Moreover, several docking-based studies on *Artemisia*-derived flavonoids have reported high predicted binding affinities toward DNA gyrase, EGFR, and urease, consistent with the interaction patterns and predicted binding energies observed in the current work. Collectively, these parallels reinforce the reliability of the present results and situate MEAA within the broader pharmacological profile reported for related *Artemisia* species. These findings also reinforce the potential of *Artemisia* species as sustainable plant bioresources for future biobased and pharmaceutical applications.

### Limitations

This study has several limitations. The biological activities were evaluated using in vitro models and a crude methanolic extract rather than isolated compounds; therefore, the observed effects may reflect synergistic interactions among multiple phytoconstituents. In addition, molecular docking was employed to provide theoretical insights into potential target–ligand interactions and does not substitute for experimental validation. Consequently, further studies involving compound isolation, pharmacokinetic evaluation, in vivo models, and additional antioxidant assays such as ABTS and hydroxyl radical scavenging assays are required to further validate the therapeutic relevance of these findings.

## Conclusion

The present study aimed to identify the chemical constituents of the MEAA and to evaluate its antioxidant, antimicrobial, cytotoxic, and molecular docking properties. The phytochemical profile obtained by LC-MS in both positive and negative ionization modes revealed that MEAA is particularly rich in flavonoids, including aglycones and glycosides such as myricetin, acacetin, quercetin, naringenin, kaempferol-3-O-glucoside and apigenin-7-O-neohesperidoside. Biological evaluation showed that MEAA exhibited moderate antibacterial activity against selected Gram-positive and Gram-negative bacterial strains and demonstrated weak free-radical scavenging activity in the DPPH assay. Furthermore, MEAA displayed moderate cytotoxic activity against hepatocellular carcinoma cells (HepG-2) with an IC_50_ value of 59.2 µg/mL when compared to the reference drug Adriamycin (IC_50_ = 6.9 µg/mL). Molecular docking analysis indicated that several flavonoids, particularly myricetin, apigenin, and hispidulin, exhibited favorable binding orientations and predicted binding energies toward cell targets. Overall, this study provides experimental and computational evidence that *A. abyssinica* is a promising natural source of bioactive compounds with antioxidant, antimicrobial, and cytotoxic properties. Future investigations focusing on the isolation, purification, and biological evaluation of individual compounds are warranted to validate their specific activities and assess their therapeutic potential. Future research should also explore the in vivo efficacy and safety of the identified compounds, as well as the development of formulations for potential therapeutic applications.

## Data Availability

Data are available from the corresponding author on reasonable request.

## Abbreviations

ATCC: American Type Culture Collection.
DFT: Density Functional Theory.
DMSO: Dimethyl sulfoxide.
DPPH: 2,2-Diphenyl-1-picrylhydrazyl.
EGFR: Epidermal Growth Factor Receptor.
ESI: Electrospray ionization.
FBS: Fetal bovine serum.
GBVI/WSA dG: Generalized Born/Volume Integral with Weighted Surface Area (MOE binding free energy).
GC–MS: Gas chromatography–mass spectrometry.
HepG-2: Human hepatocellular carcinoma cell line.
IDA: Information-dependent acquisition.
IC_50_: Half-maximal inhibitory concentration.
LC–MS/MS: Liquid chromatography–tandem mass spectromtry.
MEAA: Methanolic extract of *Artemisia abyssinica*.
MBC: Minimum bactericidal concentration.
MIC: Minimum inhibitory concentration.
MOE: Molecular Operating Environment.
MS-DIAL: Mass Spectrometry-Data Independent AnaLysis.
RMSD: Root-mean-square deviation.
RPMI-1640: Roswell Park Memorial Institute medium 1640.
TIC: Total ion chromatogram.
TD-DFT: Time-dependent density functional theory.
